# Changes of Foxo3a in PBMCs and its associations with stress hyperglycemia in acute obstructive suppurative cholangitis patients

**DOI:** 10.18632/oncotarget.20011

**Published:** 2017-08-07

**Authors:** Niu Bailin, Chen Nan, Li Peizhi, He Kun, Zhu Xiwen, Ren Guosheng, Gong Jianping, Zhang Wenfeng

**Affiliations:** ^1^ Department of Emergency and Department of Intensive Care Unit, The First Affiliated Hospital of Chongqing Medical University, Chongqing 400010, P.R. China; ^2^ Chongqing Key Laboratory of Hepatobiliary Surgery and Department of Hepatobiliary Surgery, The Second Affiliated Hospital of Chongqing Medical University, Chongqing 400010, P.R. China; ^3^ Department of Anesthesia, The Second Affiliated Hospital of Chongqing Medical University, Chongqing 400010, P.R. China; ^4^ Department of Endocrine and Breast Surgery, The First Affiliated Hospital of Chongqing Medical University, Chongqing 400010, P.R. China

**Keywords:** sepsis, stress hyperglycaemia, forkhead box O3a, nuclear factor κB, Akt

## Abstract

**Objective:**

The levels of Foxo3a in the peripheral blood mononuclears cells (PBMCs) before and after treatment were detected in acute obstructive suppurative cholangitis (AOSC) patients to evaluate the associations between Foxo3a and stress hyperglycemia (SHG).

**Methods:**

PBMCs were obtained from AOSC patients (n=28) on admission (AP), from patients at 1 week after cure (RP) and from healthy volunteers (HV) (n=14) to evaluate the relationship between the protein levels of Foxo3a and the serum levels of glucose. Signaling pathways, which link inflammation and glycometabolism, simultaneously affecting the expression of Foxo3a, were detected. In addition, cytokines were detected in PBMCs and AOSC mouse models, which were pre-treated with Foxo3a agonist.

**Results:**

The levels of glucose and p-Foxo3a in the AP were significantly higher than those in the RP and HV, where as the levels of Foxo3a in the AP were lower than those in the RP and HV. Foxo3a levels in the AP normalized against RP were strongly negatively correlated with the glucose levels in the AP normalized against RP. The levels of sphingosine-1-phosphate receptor 2 (S1PR2) in the AP were higher than those in the RP and HV. In addition, inhibition of Foxo3a phosphorylation, coupled with the down-regulation of S1PR2, attenuated the LPS-induced inflammatory response in the PBMCs and AOSC mouse models.

**Conclusions:**

Foxo3a is correlated with the dysregulation of glucose homeostasis in the pathogenesis of AOSC-induced sepsis by inhibiting the activation of PI3K/Akt-S1PR2 and NF-κB pathways, hinting at a switched role and therapeutic potentialities in the early stage of sepsis.

## INTRODUCTION

Acute obstructive suppurative cholangitis (AOSC) is one of the most important and direct causes of deaths in patients with biliary tract diseases [1–2]. Mechanical obstructions, such as biliary calculi, biliary ascariasis, and biliary tract tumor, block up the common bile duct, leading to suppurative infection and empyema; high biliary pressure leads to a large number of bacteria and lipopolysaccharide (LPS) translocation into the peripheral blood, further resulting in sepsis, which can cause a series of serious complications, such as septic shock and multiple organ failure [3–4]. Stress hyperglycemia (SHG), is believed to be associated with poor outcomes of AOSC, is usually accompanied by sepsis in the eruption stage because of severe dysregulation of body homeostasis and rapid secretion of pro-inflammatory cytokines [5–6]. In addition, bacteria and LPS translocate into the peripheral blood, further activating downstream nuclear factor κB (NF-κB) via Toll-like receptors (TLRs) producing a large number of pro-inflammatory cytokines, such as tumor necrosis factor α (TNF α) [7–8]. Thus, the activation of NF-κB is link to the pathogenesis of sepsis.

Generally, the activation of NF-κB first requires phosphorylation and degradation of inhibitor of NF-κB α (IκB-α), which is an anchor on NF-κB polymers [8–9]. NF-κB polymers are activated and enter the cell nucleus as soon as IκB-α is degraded, thus further promoting the transcription and expression of pro-inflammatory cytokines [10–11]. Therefore, inhibiting the phosphorylation of IκB is an effective means to block the secretion of the pro-inflammatory cytokines induced by bacteria and LPS.

Forkhead box O3a (Foxo3a) not only directly phosphorylates IκB but also functions in the activation of NF-κB pathway in the infection disease [12–13]. In addition, it is well known that the phosphatidyl iositol 3-kinase (PI3K)/Akt/Foxo3a axis plays a key role in the regulation of glucose uptake and metabolism. PI3K/Akt activation is essential for the degradation of IκB-α and the activation of NF-κB by sphingosine-1-phosphate (S1P) via sphingosine-1-phosphate receptor 2 (S1PR2) [14–15]. However, few studies have reported the roles of Foxo3a in the relationship with glucose homeostasis in AOSC-induced sepsis. Therefore, our experiment detected the expression changes in Foxo3a in the peripheral blood mononuclear cells (PBMCs) of patients with acute obstructive suppurative cholangitis (AOSC) and discussed the associations of Foxo3a and SHG in sepsis.

## RESULTS

### General condition of patients

Among all of the patients with AOSC (n=28), 24 cases had typical Charcot's triad and 18 cases had typical Reynold's pentad. Twenty-three cases had secondary choledocholithiasis caused by gallstones including 4 cases of Mirziz's syndrome, 7 cases of hepatolithiasis, 3 cases of benign bile duct stenosis and 2 cases of common bile duct bottom expansion. All of the patients underwent emergency operations such as common bile duct exploration combined with T tube drainage, bile duct puncture, cholangiojejunostomy and hepatic lobectomy, as soon as they were diagnosed with AOSC. No deaths occurred during hospitalization (5-16 days), 7 cases suffered serious complications after surgery. All of the patients achieved clinical cure criteria. The clinical presentations and laboratory results of the patients were shown in Tables 1 and 2, respectively.

**Table 1:** The clinical presentation and management of patients

Presentation	No.	Concomitant disease	No.	Management	No.	Complication	No.
fever/chill	25	cardiac insufficiency	2	drainage of common bile duct	13	pneumonia	4
Jaundice	24	pulmonary insufficiency	1	bile duct puncture	4	effusion and infection of liver cutting surface	1
epigastric pain	27	renal insufficiency	1	cholangiojejunostomy	6	bile leakage	2
cachexia	18	cirrhosis combined with HBV	3	subtotal gastrectomy	17		
lethargy/coma	18	a history of biliary disease	21	hepatic lobectomy	5		
nausea/vomiting	15	a history of biliary surgery	7				
abdominal distension	11						
diarrhea	12						

**Table 2:** Characteristics of AOSC patients and healthy volunteers

	AOSC		HV	χ^2^ value	p value
**Count**	28		14	-	-
**Age** (years)	38.46±13.32		36.86±9.59	0.136	0.691
**Gender** (male/female)	12/16		8/6	0.764	0.382
**Clinical index**	**AP**	**RP**	**HV**	**χ^2^ value**	**p value**
**WBC** (×10^9^/L)	16.37±2.87	9.22±1.99*	8.14±1.64**	0.07	* 0.00** 0.00
**CRP** (mg/L)	66.24±21.25	6.28±2.22*	5.26±1.61**	0.00	* 0.00** 0.00
**Procalcitonim** (ng/mL)	0.96±0.36	0.03±0.02*	0.03±0.01**	0.00	* 0.00** 0.00
**Creatinine** (umol/L)	168.11±28.51	78.24±19.70*	68.05±8.55**	0.00	* 0.00** 0.00
**BUN** (mmol/L)	12.87±2.41	4.90±1.48	4.81±1.11	0.01	* 0.00** 0.00
**TBIL** (umol/L)	141.96±39.31	13.52±3.88*	9.66±4.29**	0.00	* 0.00** 0.00
**ALT** (U/L)	197.09±41.46	25.52±7.53	20.45±9.40	0.00	* 0.00** 0.00
**AST** (IU/L)	212.02±38.24	23.87±8.78	20.94±7.79	0.00	* 0.00** 0.00
**ALP** (IU/L)	569.32±214.04	124.29±36.60*	101.07±34.33**	0.00	* 0.00** 0.00
**γ-GGT** (U/L)	119.08±26.48	28.08±14.41	21.02±10.45	0.00	* 0.00** 0.00

*, RP *vs* AP; **, HV *vs* AP.

### Changes in glucose, LPS, TNF α and IFN γ

**Figure 1:**
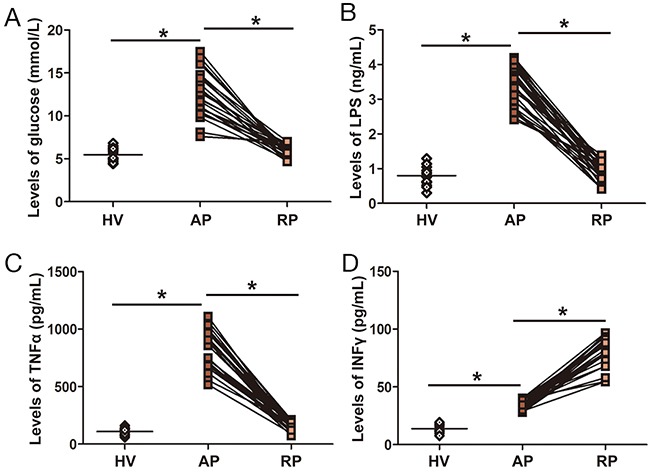
Serum levels of glucose, LPS, TNF α and INF-γ in AOSC patients **(A)** Serum levels of glucose in the AP were significantly higher than those in the RP and HV. **(B)** Serum levels of LPS in the AP were significantly higher than those in the RP and HV. **(C)** Serum levels of TNF α in the AP were significantly higher than those in the RP and HV. **(D)** Serum levels of INF-γ in the AP were significantly lower than those in the RP, but higher than those in HV. *, *p*<0.05.

The serum levels of glucose, LPS and TNF α in the acute phase (AP) were significantly higher than those in healthy volunteers (HV) and decreased to normal levels after surgery and anti-inflammation therapy (recovery phase (RP)) (Figure 1 and Supplementary Table 1). The levels of interferon γ (IFN γ) in the AP were higher than those in HV, and the levels of IFN γ in the RP were lower than those in the AP, but they were still higher than in the HV (Figure 1 and Supplementary Table 1). The data showed that LPS infiltrated from the intestine to the circulation in the eruption stage of infection, resulting in an imbalance in and pro- and anti-inflammatory cytokines cytokines.

### Changes in expression of Foxo3a in PBMCs

**Figure 2:**
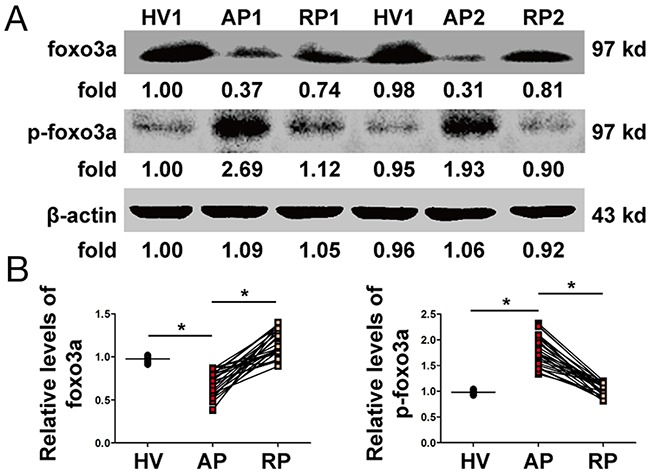
Changes in Foxo3a in PBMCs **(A)** The expression and phosphorylation levels of Foxo3a were tested by WB in two patients before (AP) and after (RP) treatment and in one healthy volunteer (HV). **(B)** The protein levels of Foxo3a in AOSC patients in the AP were significantly lower than those in HV and were increased to normal levels after treatments, whereas the expression of p-Foxo3a increased dramatically in the AP in AOSC patients compared to those in the RP and HV. *, *p*<0.05.

**Figure 3:**
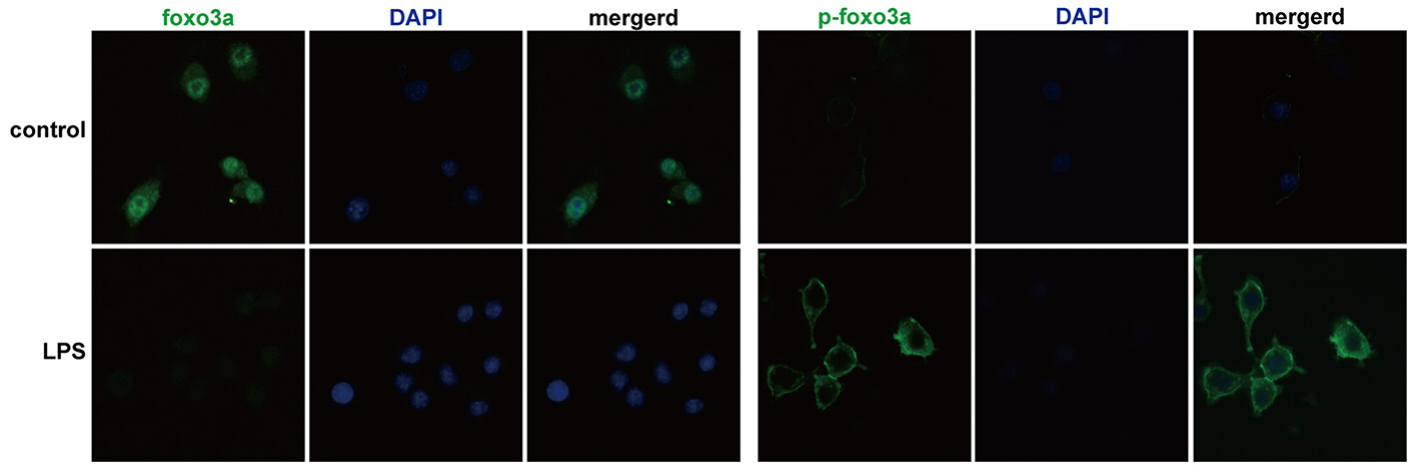
Foxo3a and p-Foxo3a levels in PBMCs were detected by immunofluorescence assay Foxo3a was phosphorylated and discharged into the cytoplasm after stimulation by a lethal dose of LPS (100 ng/mL) (fluorescence microscope, FITC, 400×).

The protein levels of Foxo3a in AOSC patients in the AP were significantly lower than those in HV and increased to normal levels after treatment (Figure 2 and Supplementary Table 2). However, the expression of p-Foxo3a dramatically increased in the AP in AOSC patients, compared to those in the RP and HV (Figure 2 and Supplementary Table 2). In addition, Foxo3a was decreased in nuclei and p-Foxo3a increased around the nuclei of PBMCs after treatment with a lethal dose of LPS (Figure 3). These results showed that Foxo3a was involved in the early stage of AOSC-induced sepsis.

### Activation of the NF-κB and PI3K/Akt pathways in PBMCs

**Figure 4:**
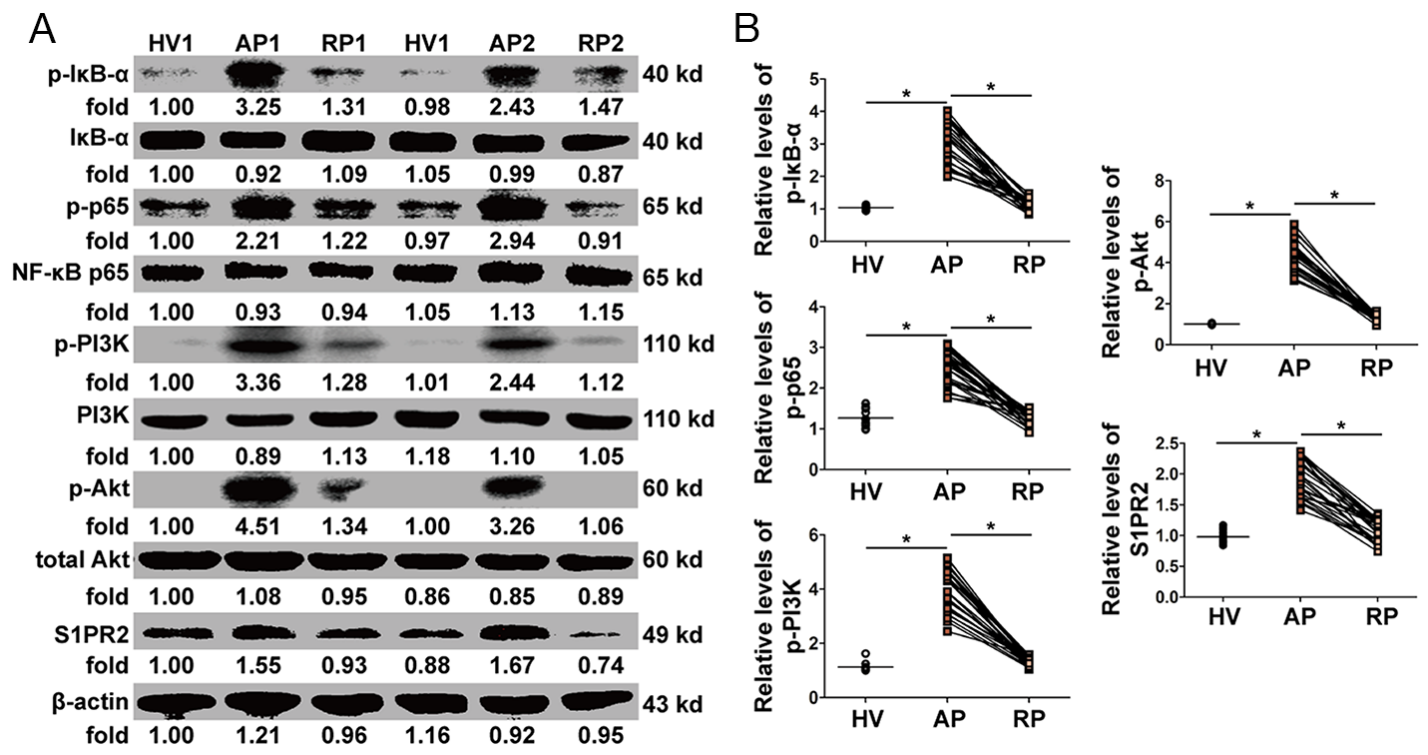
Activation of NF-κB and PI3K/Akt pathways in PBMCs **(A)** The expression and phosphorylation levels of Foxo3a were tested by WB in two patients before (AP) and after (RP) treatment and in one healthy volunteer (HV). **(B)** The protein levels of p-IκB-α and p-NF-κB p65 increased dramatically in the AP in AOSC patients and decreased to normal levels after treatment, while the expression of p-PI3K, p-Akt and S1PR2 dramatically decreased after treatment. *, *p*<0.05.

The protein levels of p-IκB-α and p-NF-κB p65 dramatically increased in the AP in AOSC patients and reduced to normal levels after treatment (Figure 4 and Supplementary Table 3), whereas the expression levels of p-PI3K, p-Akt and S1PR2 returned to thier former unexcited state (Figure 4 and Supplementary Table 3). The data showed that the NF-κB and PI3K/Akt pathways were involved in the pathogenesis of AOSC-induced sepsis.

### The relationship between Foxo3a and S1PR2 in PBMCs

**Figure 5:**
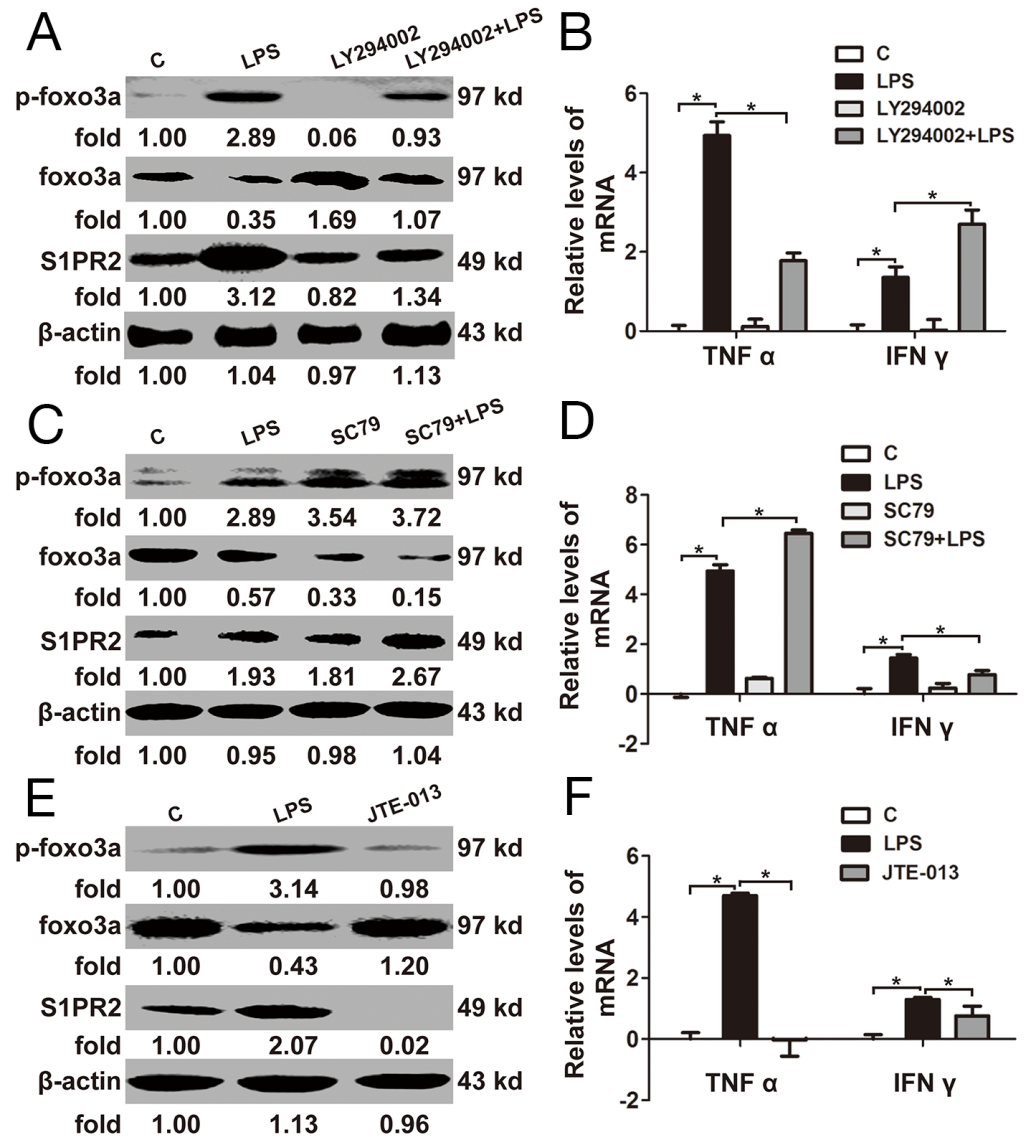
The relationship between Foxo3a and S1PR2 in PBMCs **(A)** and **(B)** PBMCs were pre-treated with LY294002 for 1 h before stimulation with LPS for 2 h: the protein and phosphorylation levels of Foxo3a and S1PR2 were tested by WB assay; the mRNA levels of TNF α I and IFN γ were detected by RT-PCR. **(C)** and **(D)** PBMCs were pre-treated with SC79 for 1 h before stimulation with LPS for 2 h: the protein and phosphorylation levels of Foxo3a and S1PR2 were tested by WB assay; the mRNA levels of TNF α and IFN γ were detected by RT-PCR. **(E)** and **(F)** PBMCs were treated with LPS and JET-013 for 2 h: the protein and phosphorylation levels of Foxo3a and S1PR2 were tested by WB assay; the mRNA levels of TNF α and IFN γ were detected by RT-PCR. *, *p*<0.05.

Foxo3a protein levels were increased by PI3K inhibitor (LY294002) in PBMCs. S1PR2 and TNF α induced by LPS were resisted by LY294002, while IFN γ were increased by LY294002 in PBMCs (Figure 5A and 5B). At the same time, Foxo3a protein levels were inhibited by Akt agonist (SC79) in PBMCs. S1PR2 and TNF α induced by LPS were increased by SC79, whereas IFN γ were decreased by SC79 in PBMCs (Figure 5C and 5D). Further, to validate the regulatory relation between Foxo3a of S1PR2, an inhibitor of S1PR2 (JTE-013) was added into PBMC in vitro. The protein levels of S1PR2 were decreased by JTE-013, while the protein and phosphorylation levels of Foxo3a did not show any changes (Figure 5E). Similarly, the mRNA levels of TNF α and IFN γ did not show any changes when PBMCs were stimulated by JTE-013 (Figure 5F). These results showed that inhibition of Foxo3a phosphorylation attenuated the LPS-induced inflammatory response by decreasing the expression of S1PR2.

### Correlation between of Foxo3a and SHG

**Figure 6:**
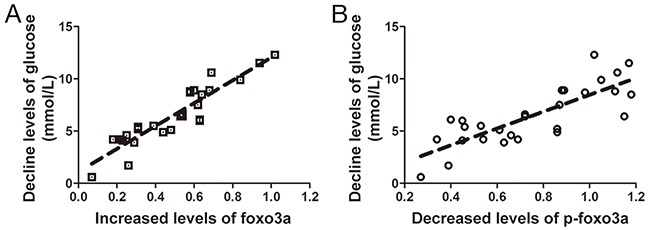
Correlation between expression and phosphorylation levels of Foxo3a and levels of glucose **(A)** Foxo3a levels in the RP normalized against the AP were strongly positively correlated with the glucose levels in the AP normalized against the RP (r^2^=0.845). **(B)** p-Foxo3a levels in the AP normalized against the RP were positive relates with the glucose levels in the AP normalized against the RP (r^2^=0.637).

Foxo3a levels in the AP normalized against the RP were strongly negative correlated with glucose levels in the AP normalized against the RP, while p-Foxo3a levels in the AP normalized against the RP were positively correlated with glucose levels in the AP normalized against the RP (Figure 6). The data showed that the Foxo3a decrease was likely correlated with hyperglycemia in AOSC patients.

### Effect of Foxo3a in an AOSC mouse model

**Figure 7:**
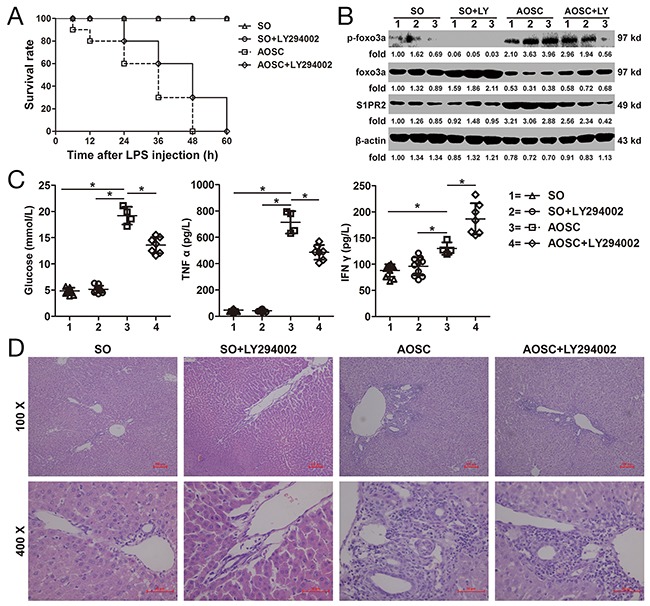
Effect of Foxo3a on AOSC mouse model **(A)** Survival time of mice in the SO, SO+LY294002, AOSC and AOSC+LY294002 groups were observed at 6, 12, 24, 48 and 60 h after LPS injection. **(B)** The protein and phosphorylation levels of Foxo3a and S1PR2 were tested by WB assay in peritoneal cells in the SO (n=10), SO+LY294002 (n=10), AOSC (n=4) and AOSC+LY294002 (n=4) groups at 24 h after LPS injection. **(C)** The serum levels of glucose, TNF α and IFN γ were tested by ELISA in the SO (n=10), SO+LY294002 (n=10), AOSC (n=4) and AOSC+LY294002 (n=4) groups at 24 h after LPS injection. **(D)** The HE staining of liver tissue in the SO (n=10), SO+LY294002 (n=10), AOSC (n=4) and AOSC+LY294002 (n=4) groups at 24 h after LPS injection, (100× and 400×). *, *p*<0.05.

The survival time of mice in the AOSC group was shorter than that in the AOSC+LY294002 group (Figure 7A). However, the overall survival rate showed no statistically difference between the AOSC group and the AOSC+LY294002 group. Similarly, clinical indices, such as C-reactive protein (CRP), procalcitonin, creatinine, urea nitrogen (BUN), total bilirubin (TBIL), alanine transaminase (ALT), aspartate aminotransferase (AST), alkaline phosphatase (ALP), and glutamyltranspeptidase (γ-GGT), in the peripheral blood of the AOSC group showed no differences as compared with those in the AOSC+LY294002 group (Table 3) after modeling of 24 h. At the same time, serum levels of glucose and TNF α in the AOSC group were significant higher than those in the AOSC+LY294002 group, while the levels of IFN γ were significantly lower than those in the AOSC+LY294002 group (Table 3 and Figure 7C). Similarly, the levels of p-Foxo3a and S1PR2 in the peritoneal cells of the AOSC group were significantly higher than in the AOSC+LY294002 group, while the protein levels of Foxo3a were significantly lower than those in the AOSC+LY294002 group (Figure 7B). However, necrotic areas, inflammatory cells infiltration and biliary fibrosis were clearly observed on histological examination of liver tissues in both the AOSC and AOSC+LY294002 groups (Figure 7D). These results indicated that inhibition of the phosphorylation of Foxo3a attenuated glucose levels and prolonged survival time by down-regulating the expression of SP1R2 to inhibit the secretion of pro-inflammatory cytokines in peritoneal cells in AOSC mouse models, although it had nothing to do with the mice's overall survival rate or physiological indices.

**Table 3:** Clinical indices in mouse model of AOSC

Index	SO (n=10)	SO+ LY294002 (n=10)	AOSC (n=4)	AOSC+ LY294002 (n=7)
CRP (mg/L)	2.81±0.66*	3.26±0.67**	48.14±11.32	40.07±16.34
Procalcitonim (ng/mL)	0.35±0.09*	0.33±0.06**	2.52±0.20	2.08±0.26
Creatinine (umol/L)	47.74±4.25*	45.18±4.48**	167.65±19.20	162.75±28.80
BUN (mmol/L)	6.18±1.05*	6.15±1.12**	21.97±5.11	23.39±3.57
TBIL (umol/L)	10.59±2.97*	10.07±3.14**	160.75±29.54	170.83±32.51
ALT (U/L)	27.00±6.01*	28.71±6.90**	290.07±38.66	285.12±71.08
AST (IU/L)	97.65±8.31*	104.86±8.78**	1052.77±180.85	1006.19±132.00
ALP (IU/L)	108.98±13.39*	111.02±12.48**	1550.83±181.53	1765.73±330.16
γ-GGT(U/L)	20.06±4.13*	19.05±4.88**	94.71±11.74	100.59±11.38
Glucose (mmol/L)	4.85±0.62*	5.17±0.68**	19.20±1.68	13.59±1.54***
TNF α (pg/mL)	46.73±8.84*	42.87±8.42**	713.83±85.06	486.10±56.05***
IFN γ (pg/mL)	87.93±11.87*	95.93±17.34**	130.06±11.80	186.62±29.73***

*, SO *vs* AOSC, *p*<0.05; **, SO+ LY294002 *vs* AOSC, *p*<0.05; ***, AOSC+ LY294002 *vs* AOSC, *p*<0.05.

## DISCUSSION

In this experiment, the variation in Foxo3a in PBMCs before and after treatment of AOCS patients was consistent with changes in LPS and inflammatory cytokines via the activation of NF-κB and PI3K/Akt. Inhibition of Foxo3a phosphorylation reduced the LPS-induced inflammation by down-regulating the expression of S1PR2 both in the PBMCs and in AOCS mouse models. In addition, the Foxo3a decrease was likely related to hyperglycemia in AOSC patients. Thus, Foxo3a was a negative regulatory factor in the pathogenesis of AOCS-induced sepsis.

As we known, SHG induced by glucocorticoids and catecholamine is a typical complication of sepsis and is closely related to prognosis [16–17]. Larger amounts of pro-inflammatory cytokines, such as TNF α evoked by LPS, not only induce DNA damage in β cells by directly promoting the expression of cyclic guanosine monophosphate (cGMP) but also inhibit the secretion of insulin by inducing the synthesis of inducible nitric oxide synthase (iNOS) [18–19]. In addition, TNF α blocks insulin signaling and glucose transport by inhibiting the tyrosine phosphorylation of insulin receptor substrates (IRS-1) and decreasing the expression of glucose transporter 4 (GLUT-4) [20]. Increased levels of TNF α and glucose were observed both in the immunocytes of AOSC patients and mouse models in this study. Thus, uncontrolled release of pro-inflammatory cytokines induced by LPS is a major factor that leads to insulin resistance and SHG.

The NF-κB pathway is one of the main mechanisms for secreting large numbers of pro-inflammatory cytokines, such as TNF α, worsening the systemic inflammatory response in sepsis [21–22]. In addition, levels of IFN γ returned to relatively low levels after cure; however, the levels of IFN γ in the RP remained slightly higher than those in HV. IFN γ was reported to restore partially aerobic glycolysis and to decrease mortality during the late phase of sepsis [23]. SHG is a result of an imbalance in pro- and anti-cytokines in the cytokine storm phase, in which the immune systems are disabled and immunocytes shift their glucose metabolism patterns [24–25]. Shang et al. asserted that p-Foxo3a phosphorylates IκB-α to activate NF-κB pathway when macrophages are irrigated by LPS [26]. Thompson et al. insisted that Foxo3a resists the nuclear import of NF-κB, while phosphorylated Foxo3a directly activates NF-κB to promote the secretion of TNF α when pro-inflammatory cells are stimulated by bacteria or LPS [27]. In addition, phosphorylation of Foxo3a was affected by PI3K/Akt activation caused by infection or higher levels of blood glucose [28]. The data in this study also showed that PI3K inhibitor restrained Foxo3a phosphorylation to decrease the secretion of TNF α in both the PBMCs and AOSC mouse models. Thus, at least in this experiment, the Foxo3a decrease worsened the SHG in AOSC patients.

Foxo3a is one of the transcription factors in the forkhead box family, as well as a downstream molecule of the PI3K/Akt signaling pathways [29–30]. Phosphorylated Foxo3a escapes from DNA-binding sites and discharges into the cytoplasm to reduce its transcriptional activity as soon as the PI3K/Akt pathway is activated by exogenous irritants [31]. In addition, Foxo3a inhibits the generation of pro-inflammatory cytokines, such as TNF α and IL-6 in LPS-induced PBMCs [32]. Under a lethal dose of LPS stimulation, the expression of Foxo3a in PBMCs declined sharply, along with increased levels of TNF α in PBMCs [33]. Consistently, in this study, the Foxo3a decrease is not only caused by LPS translocation, but it also resulted in hyperglycemia induced by an uncontrolled release of pro-inflammatory cytokines. Thus, Foxo3a alleviates SHG in AOSC-induced sepsis by damping a cytokine storm.

Another cellular messenger, S1PR2, is the most important receptor for S1P in macrophages and was positively correlated with the severity of sepsis [34–35]. S1PR2 has been reported to induce NF-κB activation by promoting the phosphorylation of PI3K/Akt [36–37]. Cui et al. reported that S1PR2 protects against anaphylactic shock by inhibiting the synthesis of endothelial nitric oxide [38]. Yang et al. asserted that S1PR2/3 accelerates liver injury in mouse models of bile duct ligation by promoting the motility of bone marrow-derived macrophages and inspiring pro-inflammatory cytokines by activating the pertussis toxin/PI3K/Rac1 signaling pathway [39]. In our study, S1PR2 in the AP was significantly higher than in the RP and HV. Suppression of Foxo3a phosphorylation down-regulated the expression of S1PR2 in PBMCs, while agitation of Foxo3a phosphorylation up-regulated the expression of S1PR2 in PBMCs. In addition, the expression of S1PR2 in peritoneal immune cells was inhibited by LY294002 which is an inhibitor of Foxo3a phosphorylation, leading to lower levels of TNF α and glucose, although these outcomes were not related to the overall survival of AOSC mouse models. Thus, this study is the first time to report that Foxo3a is a negative regulator of S1PR2 for inspiring AOSC-induced sepsis.

In conclusion, Foxo3a was correlated with the dysregulation of glucose homeostasis in the pathogenesis of AOSC-induced sepsis by inhibiting activation of the PI3K/Akt-S1PR2 and NF-κB pathways, hinting at a switched role and therapeutic potentiality in the early stage of sepsis.

## MATERIALS AND METHODS

### Patients

All of the patients with AOSC were diagnosed and hospitalized in the Second Affiliated Hospital of Chongqing Medical University from January 2015 to June 2016. Diagnosis was subject to magnetic resonance cholangiopancreatography (MRCP) or computer tomography (CT) combined with clinical manifestations and serological test results. Patients were jointly diagnosed by 3 doctors with more than 20 years of clinical experience in hepatobiliary surgery, based on Tokyo Guidelines [40]. All of the patients routinely received cefotiam hydrochloride (1.0 g), polyene phosphatidyl choline (465 mg) and glutathione (1.2 g) twice per day for 3-5 d after surgery. The inclusion, exclusion and recovery criteria are shown in Table 4.

**Table 4:** The inclusion, exclusion and recovery criteria

**Inclusion criteria**
Diagnosed and hospitalized at the Second Affiliated Hospital of Chongqing Medical University.
Aged between 18-70 years old.
Patients who had the pathological basis of cholangitis and common bile duct obstruction with operation indications of common bile duct drainage based on Tokyo Guidelines.
Clinical manifestation conforms to the guidelines for management of severe sepsis and septic shock [46].
**Exclusion criteria**
Patients conform to the inclusion criteria but refused to experimental arrangement.
Patients were older than 70 or younger than 18 year old.
AOSC was caused by tumor or congenital sclerosing cholangitis.
Patients have received anti-infective therapy or relieved biliary obstruction before admission.
Patients have surgical contraindications or major organs disease such as severe heart diseases, pneumonosis, renal failure that cannot tolerate the biliary puncture, biliary tract surgery or local and general anesthesia.
Patients had a history of diabetes or had been diagnosed for diabetes during hospitalization.
**Criteria for Recovery**
Biliary obstruction was completely relieved.
Hematological index return to normal levels.
Clinical symptoms of biliary obstruction or sepsis completely faded.

### Healthy volunteers

Volunteers were recruited from regular checkups at the Second Affiliated Hospital of Chongqing Medical University from January 2014 to June 2016. People with tumors or infectious diseases or who did not agree with our experimental arrangement was excluded. Healthy volunteers and patients were matched at a 1:2 ratio.

### Ethics, consent and permissions

All of the recruited participants including AOSC patients and healthy volunteers were well informed of our experimental plans. All of the recruited participants agreed to our experimental arrangement, and all participants with informed consent donated their blood for this study. In addition, this study was approved by the Ethics Committee of the Second Affiliated Hospital of Chongqing Medical University. All of the operations for AOSC patients and healthy volunteers in this study strictly conformed to the World Medical Association's Declaration of Helsinki.

### Separation of PBMCs

A total of 25 ml peripheral blood was collected from each AOSC patient as soon as admission (AP) and 1 week after cure (RP). The serum was separated from samples by density gradient centrifugation (2000 r/min, 10 min) for enzyme linked immunosorbent assay (ELISA) detection. The residual blood cells were mixed with an equal volume of phosphate buffer solution (PBS) (0.01M, pH=7.4) and were placed in a centrifuge tube; then, an equal volume of peripheral blood separation liquid (LDS1075, Jingyang, Tianjin China) was gently added in the mixture and allowed to stand for 5 min before horizontal density gradient centrifugation (400 g, 20 min). The mononuclear cells were extracted after centrifugation and washed with 10 ml of PBS twice (300 g, 10 min). Finally, the obtained PBMCS were extracted for total protein. Each healthy volunteer underwent collection of a total of 25 ml of blood as soon as admission (HV), which handled with the aforementioned procedures.

### Cultivation and treatment of PBMCs

PBMCs separated from healthy volunteers with the above procedures were cultured with medium containing 10% fetal bovine serum in an incubator under conditions of 37°C, 5% CO_2_ and appropriate humidity. After 6 h of cultivation, the medium was replaced to remove the non-adherent cells. The adherent cells continued to cultivate for 12 h. Then, PBMCs were pretreated with LY294002 (30 μM) (S1105, Selleck, USA) and SC79 (4 μg/ml) (S7863, Selleck, USA) for 1 h, followed by treatment with LPS (100 ng/ml) (L5293, SIGMA, USA) for 2 h. For S1PR2 inhibitor testing, PBMCs were pretreated with JET-013 (1 μM) (S7182, Selleck, the USA) and LPS (100 ng/ml) for 2 h. The total protein and RNA of PBMCs were collected for western blotting (WB) and real-time polymerase chain reaction (RT-PCR). Safe doses of LY294002, SC79 and JET-013 were verified in previous studies [39, 41–42].

### Immunofluorescence assay

The PBMCs were grown on slides pre-treated with LPS (100 ng/ml) for 2 h and were treated with 0.1%Triton (ST795, Beyotime, China) for 10 min at room temperature after being fixed using 4% paraformaldehyde at 4 °C, following by blocking with 5% goat serum for 1 h at room temperature. The cells were stained with primary antibody at 4 °C overnight and then were incubated with secondary antibodies labeled with fluorescein isothiocyanate (FITC) for 60 min at room temperature, followed by 4,6-diamidino-2-phenylindole dihydrochloride (DAPI) for nuclear staining for 2 min. Then, the slides were observed using a fluorescence microscope in dark phase with a 488 nm radial. The antibodies used in the assay are showed in Supplementary Table 4.

### Animals and AOSC mouse models

Male C57BL/6 mice (pathogen-free, 20-22 g, 8 weeks old) were purchased from the Experimental Animal Center of Chongqing Medical University (Chongqing, China). All surgeries on the mice obtained permission from the Ethics Committee of Chongqing Medical University. The mice received good, humane care before and after modeling.

The AOSC mouse models were created according to previous reports with slight changes [43]. Briefly, after an overnight fast, the mice were first anesthetized, and the common bile duct was dissociated and ligated. Then, the mice underwent cholecystostomy by an epidural catheter fixed in the backs of the mice. LPS in a volume of 0.5 ml (10 mg/kg) was injected immediately into the gallbladder through the epidural catheter. Then, the epidural catheter was sealed, and the mice were observed until mortality after injection of LPS. All of the surgeries were performed under 40 mg/kg 1% pentobarbital sodium salt to minimize suffering.

### Experimental groups

For survival testing, 40 mice were randomly divided into 4 groups: the sham-operation group (SO group), in which the mice received simple laparotomy surgery; the sham-operation combined with LY294002 treatment group (SO+LY294002 group), in which the mice were intraperitoneally pre-injected with LY294002 (5 mg/kg) for 6 h before laparotomy surgery; the AOSC mouse model group (AOSC group), in which the mice were modeled for AOSC using the above procedures; and the AOSC mouse model combined with LY294002 treatment group (AOSC+LY294002 group), in which the mice were pre-treated with intraperitoneal injection of LY294002 (5 mg/kg) for 6 h before submission to a AOSC model. All of the mice were observed for their survival state after surgery at 6, 12, 24, 48 and 60 h. The safe and effective dose ranges of LY294002 were previously determined [44].

Another 40 mice were randomly divided into 4 groups as described above. The mice were sacrificed to collect peritoneal cells, serum and liver tissue at 24 h after surgery. The numbers of surviving mice in the SO, SO+LY294002, AOSC and AOSC+LY294002 groups were 10, 10, 4 and 7, respectively.

### Isolation of peritoneal cells

Peritoneal cells were isolated according to the previous report [45]. Briefly, the mice were first euthanized, and the peritoneum was exposed immediately after abdominal disinfection. PBS in a volume of 5 ml was injected into the peritoneal cavity, and the peritoneum was gently massaged for 5 min. The fluid was collected as much as possible, following by centrifugation at 1500 rpm for 8 min, the residual cells were extracted for total protein.

### Western blotting assay

Total proteins were extracted from 1×10^6^ cells with 100 μl of RIPA (AR0105, Boster, Wuhan China) lysis buffer. The concentration of protein was tested using a BCA kit (AR0146, Boster, Wuhan China) and 50 μg of total protein were separated by 10% SDS-PAGE (100 V, 120 min) and then were blotted to polyvinylidene fluoride (PVDF) membranes (250 mA, 90-60 min). Bovine serum albumin at a concentration of 5% was used to block the PVDF membranes for 90 min at room temperature. Then the membranes were incubated with primary antibodies over night at 4 °C, followed by incubation with horseradish peroxidase conjugated anti-IgG secondary antibodies at 37 °C for 1.5 h. Finally, the PVDF membranes were visualized using the Chemico-EQ system (Bio-Rad, USA). The values of targeted protein bands were detected using Image Lab 4 software (Bio-Rad, USA) and were normalized against β-actin values. The antibodies used in the WB assay are shown in Supplementary Table 4.

### RT-PCR analysis

Total RNA was extracted from 1×10^6^ PBMCs using an ultrapure RNA kit (CW0597, cwbiotech, China). The RNA samples were reverse transcribed into cDNA using a Primescript™ RT Reagent Kit with gDNA Eraser (RR047A; Takara Biotechnology, Japan), strictly according to the protocols of the manufacturer. The relative expression levels of the target genes were determined using the -Delta Delta C(T) method after normalizing against the GADPH gene. The primers were showed at Supplementary Table 4.

### ELISA

The serum levels of LPS (xy-0784E, Xinyu, Shanghai), TNF α (EK0525, Boster, Wuhan China) and IFN γ (KHC4021, Thermo Fisher, the USA) were quantified using commercially available ELISA kits. All of the operations were in strict accordance with the manufacturer's instructions.

### Statistical analysis

All of the measured data in this study were subject to normal distributions, which were analyzed by the Kolmogorov-Smirnov test. Data were expressed as x¯±s and were analyzed with SPSS software (version 18.0). Data from multiple groups were compared with ANOVA, and two independent samples underwent the applied comparative t-test to perform statistical analysis. Categorical data were analyzed using the Chi-Square test. To test for correlations, Pearson's simple correlation coefficient or Spearman's rank correlation coefficient was applied. p values less than 0.05 were considered to indicate statisticallysignificant differences.

## SUPPLEMENTARY MATERIALS FIGURES AND TABLES





## Abbreviations

ALP: alkaline phosphatase
ALT: alanine transaminase
AOSC: acute obstructive suppurative cholangitis
AOSC group: AOSC mice models group
AOSC+LY294002 group: AOSC mice models combined with LY294002 treatment group
AP: acute phase
AST: aspartate aminotransferase
BUN: blood urea nitrogenc
GMP: cyclic guanosine monophosphate
CRP: C-reactive protein
CT: computer tomography
DAPI: 4,6-diamidino-2-phenylindole dihydrochloride
ELISA: enzyme linked immunosorbent assay
FITC: fluorescein isothiocyanate
Foxo3a: forkhead box O3a
GADPH: glyceraldehyde-3-phosphate dehydrogenase
γ-GGT: glutamyltranspeptidase
GLUT-4: glucose transporter 4
HV: healthy volunteers
IFN γ: interferon γ
IκB-α: inhibitor of NF-κB αi
NOS: inducible nitric oxide synthase
IRS-1: insulin receptor substrates
LPS: lipopolysaccharide
MRCP: cholangiopancreatography
NF-κB: nuclear factor κB
PBMC: peripheral blood mononuclear
PBS: phosphate buffer solution
PI3K: phosphatidyl iositol 3-kinase
PVDF: polyvinylidene fluoride
RP: recovery phase
RT-PCR: real time polymerase chain reaction
S1PR2: sphingosine-1-phosphate (S1P) via sphingosine-1-phosphate receptor 2
SHG: stress hyperglycaemia
SO group: sham-operation group
SO+LY294002 group: sham-operation combined with LY294002 treatment group
TBIL: total bilirubin
WB: western blotting
