# Supplementary material for: Changes of Foxo3a in PBMCs and its associations with stress hyperglycemia in acute obstructive suppurative cholangitis patients
Source: Oncotarget. 2017 Aug 7;8(44):76783–96. doi: 10.18632/oncotarget.20011 (PMC5652742; doi:10.18632/oncotarget.20011)
Supplement: Supplementary file 1 [file oncotarget-08-76783-s001.pdf]

Changes of Foxo3a in PBMCs and its associations with stress hyperglycemia in acute obstructive suppurative cholangitis patients

SUPPLEMENTARY MATERIALS

Supplementary Table 1: Serum levels of glucose, LPS, TNF-α and INF-γ

Index	AOSC		HV	χ2 value	*p* value
	AP	RP			
glucose (mmol/L)	12.39±2.54	5.93±0.68*	5.47±0.83**	0.000	* 0.000<br>** 0.000
LPS (ng/mL)	3.38±0.57	0.91±0.28*	0.80±0.29**	0.00	* 0.00<br>** 0.00
TNF-α (pg/mL)	840.03±166.03	153.29±39.00*	109.25±29.52**	0.00	* 0.00<br>** 0.00
INF-γ (pg/mL)	81.53±12.08	34.03±4.30*	13.75±3.63**	0.00	* 0.00<br>** 0.00

*, RP *vs* AP; **, HV *vs* AP.

Supplementary Table 2: Fold changes for p-Foxo3a and Foxo3a protein

Fold change	AOSC		HV	χ2 value	*p* value
	AP	RP			
p-Foxo3a	1.78±0.29	1.03±0.13*	0.98±0.04**	0.000	* 0.000<br>** 0.000
Foxo3a	0.68±0.14	1.17±0.15*	0.97±0.04**	0.00	* 0.00<br>** 0.00

*, RP *vs* AP; **, HV *vs* AP.

Supplementary Table 3: Fold changes for NF-κB and PI3K/Akt-S1PR2 pathway

Fold Change	AOSC		HV	χ2 value	*p* value
	AP	RP			
**p-IκB-α**	3.02±0.64	1.13±0.19*	1.05±0.06**	0.000	* 0.000<br>** 0.000
**p-p65**	2.56±0.42	1.25±0.17*	1.27±0.23**	0.00	* 0.00<br>** 0.00
**p-PI3K**	4.01±0.78	1.33±0.17*	1.07±0.04**	0.00	* 0.00<br>** 0.00
**p-Akt**	4.34±0.71	1.31±0.18*	1.00±0.03**	0.00	* 0.00<br>** 0.00
**S1PR2**	1.92±0.31	1.06±0.19*	0.98±0.10**	0.00	* 0.00<br>** 0.00

*, RP *vs* AP; **, HV *vs* AP.

Supplementary Table 4: Antibodies and primers for targeted genes

See Supplementary File 1
