# Supplementary material for: Changes of Foxo3a in PBMCs and its associations with stress hyperglycemia in acute obstructive suppurative cholangitis patients
Source: Oncotarget. 2017 Aug 7;8(44):76783–96. doi: 10.18632/oncotarget.20011 (PMC5652742; doi:10.18632/oncotarget.20011)
Supplement: Supplementary file 2 [file oncotarget-08-76783-s002.doc]

**Supplementary Table 4: Antibodies and primers for targeted genes**

**1 Antibodies**

Rabbit anti-Foxo3a antibody (12829, CST, the USA);

Rabbit anti-p-Foxo3a antibody (9466, CST, the USA);

Goat anti-p-Foxo3a antibody (34897, santa cruz, the USA);

Rabbit anti-IκB-α antibody (4812, CST, the USA);

Rabbit anti-p-IκB-α antibody (2859, CST, the USA);

Rabbit anti-NF-κB p65 antibody (4764, CST, the USA);

Rabbit anti-p-NF-κB p65 antibody (3033, CST, the USA);

Rabbit anti-AKT antibody (4691, CST, the USA);

Rabbit anti-p-AKT antibody (4060, CST, the USA);

Mouse anti-PI3K antibody (ab86714, Abcam, the USA);

Rabbit anti-p-PI3K antibody (ab182651, Abcam, the USA);

Rabbit anti-S1PR2 antibody (PA5-23208, Thermo fisher, the USA)

Mouse anti-β-actin antibody (BM0627, Boster, China);

Goat anti-rabbit IgG HRP antibody (BA1055, Boster, China);

Goat anti-mouse IgG HRP antibody (BA1051, Boster, China);

Rabbit anti-goat IgG HRP antibody (BA1060, Boster, China);

Goat anti-rabbit IgG/FITC antibody (ZF-0311, Zhongsanjinqiao, China);

Rabbit anti-goat IgG/FITC antibody (ZF-0314, Zhongsanjinqiao, China);

2 Primers for targeted genes

| Targeted genes | Primers | Length (bp) |
| --- | --- | --- |
| TNF α | Forward: 5′-GAGGCCAAGCCCTGGTATG-3′  Reverse: 5′-CGGGCCGATTGATCTCAGC-3′ | 91 |
| IFN γ | Forward: 5′-TCGGTAACTGACTTGAATGTCCA-3′  Reverse: 5′-TCGCTTCCCTGTTTTAGCTGC-3′ | 93 |
| GADPH | Forward: 5′-GGAGCGAGATCCCTCCAAAAT-3′  Reverse: 5′- GGCTGTTGTCATACTTCTCATGG-3′ | 197 |
